# Fostering childbirth education on upright positions and mobility during labor in nulliparous women

**DOI:** 10.1186/s12884-023-06166-4

**Published:** 2023-12-16

**Authors:** Hanna Borer, Ilana Dubovi

**Affiliations:** Nursing Department, Faculty of Medicine, The Stanley Steyer School of Health Professions, Tel Aviv University, Tel Aviv, 69978 Israel

**Keywords:** ICAP framework, Cognitive engagement, Childbirth education, Upright positions, Labor, Mobility

## Abstract

**Background:**

Upright labor positions and movement during labor have a positive effect on childbirth, yet the predominant labor positions are still horizontal. Therefore, it is important to explore how it is possible to improve childbirth education, particularly its instructional design, to strengthen women’s self-efficacy toward the use of upright positions and mobility during labor. The aim of the study was to evaluate the impact of an instructional approach based on a cognitive engagement ICAP (Interactive, Constructive, Active, Passive) framework on the development of knowledge, attitudes, and self-efficacy expectations toward upright positions and mobility during labor.

**Methods:**

A prospective quasi-experimental study was conducted among nulliparous women from the ultra-orthodox Jewish community (*n* = 74). While the control group (*n* = 34) participated in routine childbirth education, the intervention group (*n* = 36) learned with childbirth education that included interactive and constructive cognitive engagement activities. Participants in both groups completed a set of questionnaires regarding knowledge, attitudes, and self-efficacy.

**Results:**

The post-test analysis revealed that women in the intervention group compared to the control group gained significantly higher knowledge scores (*p* < 0.05), more positive attitudes (*p* < 0.001), and stronger self-efficacy expectations toward upright positions and mobility during labor (*p* < 0.01).

**Conclusions:**

The findings suggest that by fostering women’s cognitive engagement levels during childbirth education toward the interactive and constructive modes of the ICAP framework, women’s self-efficacy to move during labor and to use upright positions can be induced. These results can serve as a foundation to improve the overall effectiveness of childbirth instruction.

**Trial registration:**

The study was registered retrospectively.

## Background

Upright labor positions and movement during labor have demonstrated various positive effects on the process and outcomes of childbirth. In a Cochrane meta-analysis including 25 trials, upright positions were shown to be associated with a shorter first stage of labor, a reduction in cesarean birth incidence, and fewer newborn admissions to the neonatal intensive care unit [1]. Further reviews and meta-analyses have demonstrated a relation between upright labor positions and a shorter second stage of labor, a reduction in episiotomies, and fewer abnormal fetal heart rate patterns [2–7]. Moreover, a growing body of evidence reports that mobility and upright positions during labor might foster more positive childbirth experiences, perceptions of childbirth as less traumatic, and increased comfort levels [8, 9]. Building upon this evidence the World Health Organization (WHO) and other international obstetric societies have released recommendations and guidelines stating that women should be encouraged to be mobile and adopt comfortable positions of their choice, including upright positions [10, 11].

However, to date, and despite the evidence, the predominant labor and birth positions are still horizontal. A recent NHS maternity survey reported that in England on 2021 only 16% of women stated that they gave birth while standing, squatting, or kneeling [12]. This finding is in alignment with previous studies to show that mobility restrictions and horizontal positions for childbirth are still predominant worldwide [13, 14]. This gap yielded many new studies aiming to support maternal positions during labor, focusing mainly on midwives' training and healthcare system barriers [15, 16]. As a woman's self-belief and confidence in her childbirth abilities can influence both her inclinations towards certain birthing positions and her responsiveness to a midwife's encouragement for upright positions—factors that subsequently affect labor [17–20]—this study concentrates on pregnant women. Particularly, this study seeks to evaluate how it is possible to induce women's self-efficacy, attitudes, and knowledge on mobility and upright positions via childbirth education.

Formal childbirth education was developed about 80 years ago to help women play an active role in labor and be able to apply nonpharmacological approaches to cope with pain [21]. Today, the scope of childbirth education is broader and focuses on providing information about the physiology of labor, the mode of birth, management of pain, emotional issues during labor, the postpartum period, and the early weeks of motherhood. Though high-quality evidence on the outcomes of participation in childbirth education classes is still limited and inconsistent, there are some recent studies that are pointing toward childbirth classes' clear benefits. A Cochrane review analyzed 29 trials to conclude that participation in childbirth classes may reduce the rate of cesarean Sects [22]. Moreover, a recent systematic review and meta-analysis of 23 studies found that in addition to the reduced risk of a cesarean birth and the use of epidural anesthesia, childbirth education is associated also with improved childbirth self-efficacy and reduced maternal stress [23]. Importantly, Shand, Lewis‐Jones [24] in their prospective study found out that not only the attendance at childbirth education impacted birth outcomes but also the type of instruction that was provided during the classes.

Since research into the instructional design of childbirth education is scarce, the current study is grounded on the extensive body of educational research which has long recognized that instructional design plays a vital role in the learning process and learning outcomes. As the main goal of childbirth education is to empower women and their partners to play an *active role* in the co-partnership with the midwife during labor, similarly, during childbirth classes, the future parents should be also learning *actively*. “Active learning” does not necessarily require hands-on participation but is typically defined as learning that requires cognitive engagement and is often segregated into two types, “active” or “passive” [25]. However, Chi and Wylie [26] segregated active learning into four types, proposing the Interactive-Constructive-Active–Passive (ICAP) instructional framework to induce cognitive engagement.

The ICAP framework operationalizes and differentiates a four-level hierarchy of cognitive engagement that can be supported by the instruction [27] as interactive, constructive, active, or passive. Specifically, the ICAP postulates that interactive and constructive engagement levels include instructional design that supports learners’ generation of new information beyond the information that was already provided. The difference between the two is that *interactive* engagement involves co-generative collaborative behaviors such as debate or a joint dialogue with other learners. While *constructive* engagement involves individual generative behaviors, without collaborating with others, such as providing self-explanations. Interactive and constructive modes are superior to the successive modes of active engagement and passive engagement. *Active* engagement is marked by manipulating some parts of learning materials such as highlighting or underlining text; and *passive* engagement entails receiving information only, for example by listening to a lecture. Recent research, both in K-12 school instruction, higher education, and medical education, has demonstrated that instruction based on the cognitive engagement ICAP hierarchy was able to predict learning outcomes [27–29]. Building upon the effectiveness of ICAP interactive and constructive instructional modes, the present study sought to evaluate its impact on childbirth education effectiveness. Particularly, since self-efficacy is shown to be strongly related to a person's performance and behavior [30, 31], this study explores how instruction based on the ICAP framework might strengthen women's self-efficacy toward upright positions and mobility during labor.

## Methods

### Research design and participants

**Fig. 1:**
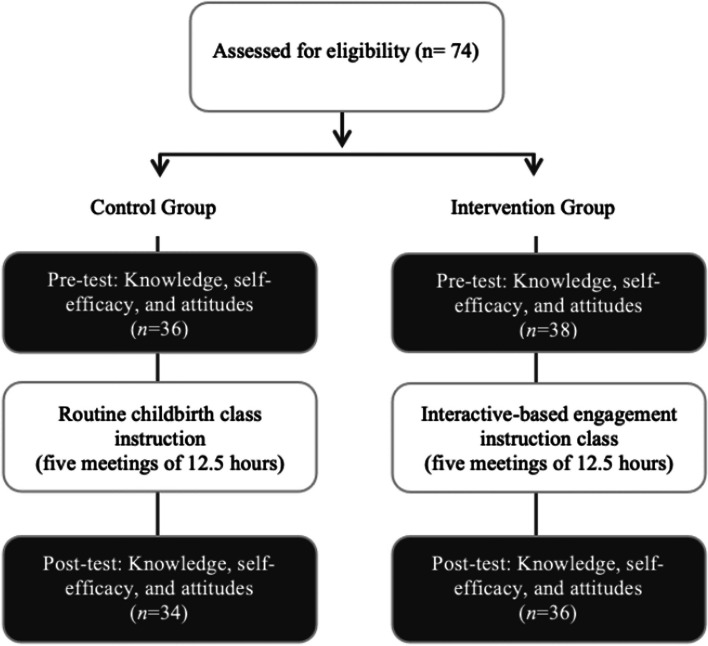
Flow chart of the study design and procedure

The study employed a quasi-experimental design, with an intervention and a control group, to examine the impact of the instructional approach to empower pregnant women to use upright positions and mobility during labor (Fig. 1). To reduce the diffusion between the intervention and control groups, and to prevent any resentful demoralization of the controls, we employed an alternating recruitment strategy which is often used in educational interventions [32, 33]. Each time a control group was filled (comprising up to 12 women), the subsequent recruitment phase would focus on populating the intervention group, and vice versa.

The inclusion criteria included nulliparous and low-risk women who participated in 8 childbirth classes and agreed to participate in the study. We referred to low-risk pregnancy as a singleton gestation absent of factors predisposing the mother, fetus, or newborn to adverse outcomes or complicated birth [34, 35]. Exclusion criteria included women who did not attend the four classes or had a high-risk pregnancy. The recruitment took place from January 2021 to March 2022. Each class was attended by 6–12 women and was funded by the local health maintenance organization. Informed consent was received from all the participants who were then assigned to one of the study’s groups. The setting was a maternity care center in an ultra-orthodox area of a city in Israel. The ultra-orthodox community comprises about 13% of the total Israeli population [36] and is characterized by strict adherence to the Jewish religion [37]. We chose to study this community due to its high demand for in-person childbirth education programs and for its particular characteristics that are suited for this study, as follows: Firstly, this community is closed and conservative with minimal exposure to online/electronic media (i.e., television, newspapers/magazines, the Internet, and smartphones). Thus, compared to non-orthodox women who are exposed to various kinds of media and online information, for ultra-orthodox women, childbirth classes are the main opportunity to get prepared for labor. Secondly, the ultra-orthodox family identity is grounded on pregnancy and birth, thus the fertility rate is high with 6.6 children on average per woman; three times the OECD average of 2.1 [36, 38]. Lastly, a growing number of ultra-orthodox women are studying and working [36, 39], which poses the childbirth educational intervention as an opportunity to empower these women even further.

The university’s ethics committee approved the study (#0001776–2).

### Research procedure

All participants were assigned either to the control group that received the routine childbirth class instruction or to the intervention group that received interactive-based engagement instruction (Fig. 1). Both groups received the same content via a childbirth class that consisted of five meetings (a total of 12.5 h) given by a nurse midwife. The course curriculum was constructed based on antenatal education standards [40, 41].

On recruitment, women in the control group received a routine instructional approach which was based on lectures, videos, discussions, and practice/rehearsals (Table 1). This routine instructional approach according to ICAP was predominantly based on a passive engagement approach. The intervention group received the same content as the control group, however, the content regarding upright positions and mobility was instructed using the ICAP constructive-interactive engagement modes (Table 1). Particularly, during the third meeting, participants in the intervention group learned with two activities based on the *discovery-learning approach* [42]. In discovery learning instead of being told or provided with the information, learners work collaboratively in pairs to construct the information by themselves via exploration and inquiry. Thus, during the first activity, to underline the importance of upright positions, women were asked to measure and compare the external transverse diameter of the pelvic outlet (the transverse diameter between the ischial tuberosities) across various labor positions [43]. Learning with this activity helped women identify which position expands the most pelvic diameters to facilitate labor. During the second activity, to represent the importance of mobility during labor, women were introduced to an elbow shape plumbing pipe and a balloon with water. The women were then asked to find the quickest and easiest way to pass the balloon through the narrow pipe. Eventually, the women noticed that the gravity and mobilization of the pipe supported the best balloon transfer. Finally, during the fourth meeting (Table 1), the women in the intervention group participated in a *role-playing debate* during which they had to defend and justify their opinions on mobility and upright positions during labor. As debating by providing arguments and contra-arguments clarifies and refines ideas, it was assumed that this learning activity would foster self-efficacy.

**Table 1:** Main topics covered by the childbirth course with two different instructional approaches toward labor positions and mobility

		Instructional approach	
Meeting #	Topics covered	Control group	Intervention group
1	• Anatomy and physiology of labor • Preparation for labor (e.g., perineal massage) • Healthy lifestyle	Lecture	
2	• The three stages of labor; how to know when you are in labor • What are the options concerning medications, assistant birth, and other interventions	Lecture & Video	
	• Comfort techniques to relieve the pain such as hydrotherapy, breathing, guided imagery, relaxation, and massage	Practicing the comfort techniques	
3	• Three principles for comfort labor: balance, relaxation, and mobility	Lecture & Videos	
	• Labor positions and mobility across labor stages	Practicing different labor positions	**Discovery-based instruction**
4	• What will happen in the hospital; a virtual visit to the delivery room	Video & Discussion	
	• Guidance to make informed decisions about labor positions and mobility; anesthesia; assistant birth; and common medical procedures	Lecture	**Role-play based learning**
5	• Breastfeeding and infant care • Early postpartum period • Personal and relationship issues	Lecture & Video	

Both the control and intervention groups completed identical questionnaires at the beginning of the childbirth course (pre-test) and again at the end of the fifth meeting (post-test). The questionnaires included one concerning sociodemographic characteristics and three others concerning respectfully self-efficacy, attitudes, and knowledge of upright positions and mobility during labor.

### Data collection instruments

#### The Childbirth Self-Efficacy Inventory (CBSEI)

This was developed by Lowe [44, 45] according to Bandura [46] self-efficacy theory, to evaluate the following two domains in relation to labor: (1) *outcome expectancy* which is defined as a belief that a given behavior will enhance coping with labor; and (2) *self-efficacy expectancy* which is defined as confidence to perform specific behaviors during labor. Following permission that was granted by the author [44], the CBSEI was translated and adapted for this study to include 13 items concerning behaviors such as relaxation, breathing exercises, and support from a companion, and 5 items concerning upright positions and mobility (e.g., “sit on the birth ball and rock your pelvis side to side”). The questionnaire was translated from English to Hebrew by a health professional using a forward-and-backward translation approach. For the outcome expectancy domain, the participants were asked to rank on a 1–10 Likert scale 18 listed behaviors for how much they can help to cope with labor. For the self-efficacy expectancy domain, the participants were asked to rank the level of their confidence in applying these same 18 behaviors during labor. The overall range of rankings for each domain ranges between 18 and 180. Cronbach’s alpha yielded a good internal consistency score of 0.89 for the outcome expectation domain and 0.93 for the self-efficacy expectancy domain, similar to previous reports [44, 47].

#### Attitudes towards upright positions and mobility during labor

This questionnaire was constructed according to Ajzen [30], which defines attitudes as the positive or negative evaluation of certain behavior. Face validity was performed by eleven expert judges (eight midwives and three faculty members). The respondents were requested to indicate their attitudes to each of 6 items on a seven-point Osgood differential semantic scale, where 1 represents negative attitudes and 7 represents positive attitudes. A sample item: “pelvic movement or rocking while dancing” with possible responses ranging from “slow down labor” to “promote progression of labor”*.* Alpha Cronbach for the entire questionnaire was 0.67, which can be considered acceptable [48].

#### Knowledge questionnaire about labor positions and mobility

A knowledge questionnaire was developed by the authors to assess the participants' knowledge of upright positions and mobility during labor. The questionnaire includes 10 multiple-choice items which were developed based on the Cochrane and systematic reviews evidence-based recommendations for women’s positions and mobility during labor [1, 3, 49]. Face validity was performed by eleven expert judges (eight midwives and three faculty members). An example item asks, “What is the best practice regarding labor dance during the first stage of labor?” The possible choices are: “Labor dancing is efficient mostly between the contractions”; “Labor dancing is efficient mostly during the contractions”; “Labor dancing is efficient between and during the contractions”; or “Labor dancing is not advisable”. Another example is: “What is true about the kneeling position?” with possible answers as follows: “It might increase the risk for cesarean delivery”; “The contractions might be more painful”; “There are fewer assisted or instrumental deliveries”; or “The length of the first labor stage is similar to horizontal positions”. Analysis of the questionnaire using Cronbach alpha yielded a good internal consistency score of 0.539, an acceptable for knowledge evaluation tools [50].

### Data analysis

The questionnaires' pre- and post-scores were analyzed using descriptive statistics (mean and standard deviation [SD]). Interaction effects between the control and intervention groups were evaluated using two-way repeated measures analysis of variances (ANOVAs). Independent sample t-tests were carried out to detect significant differences between the two groups. Finally, to evaluate associations between the participants' characteristics, knowledge, attitudes, and self-efficacy of upright positions and mobility during labor, we used a bivariate parametric correlation analysis.

Data was analyzed using SPSS (version 27, IBM Corporation, Armonk, New York).

## Results

A total of 74 nulliparous women were enrolled in the study. Of these, four women were unable to attend the childbirth classes due to pregnancy-related complications (preterm labor or bed rest). Consequently, 34 nulliparous women were assigned to the intervention group and 36 to the control group. A prior statistical power analysis was performed for sample-size estimation using G*Power software (version 3.1). With an alpha of 0.05 to detect a medium-sized effect (f = 0.25) and a power (1 − β) set on 0.80, a sample of approximately 68 participants was calculated as being satisfactory to perform repeated measures ANOVA comparisons between the two groups. Thus, our final sample size of 70 is adequate.

At the recruitment, all participants were at an advanced stage of pregnancy, on average 32 ± 3.4 weeks of gestational. Overall, the majority of women were working (96%), and the most common occupation was teaching (49%). Table 2 describes the participants' characteristics across the two study groups. To account for the possible difference in preliminary knowledge or experience between the two study groups, all participants were asked whether they ever assisted a friend during labor. The majority responded that they were never present or assisted labor, 94% from the intervention group and 100% from the control, with no significant difference (*χ*^*2*^ = 2.180, *p* = 0.14). In addition, the women were asked whether they had ever heard about mobility and upright positions during labor. Most women responded that they had heard previously about mobility and upright positions during labor, with no significant difference between intervention and control groups (94% vs. 92%, respectfully, *χ*^*2*^ = 0.158, *p* = 0.69).

**Table 2:** Comparisons between the intervention and control groups at recruitment (*n* = 70) on socio-demographic characteristics

***Variables at recruitment***	***Control (n* = *36)***	***Intervention (n* = *34)***	***Statistics***^*a*^
*Current age (years)*	22.8 ± 1.8	24.5 ± 5.2	*t* = -2.41, *p* < 0.05
*Gestational age (weeks)*	31.8 ± 3.5	32.3 ± 3.4	*t* = -0.58, *p* = 0.56
*Family income*			χ^2^ = 0.58, *p* = 0.75
*Average and above*	8 (22)	9 (27)	
*Average*	11 (31)	12 (35)	
*Less than average*	17 (47)	13 (38)	
*Education level*			χ^2^ = 9.24, *p*<0.05
*Academic degree*	3 (8)	11 (32)	
*Vocational diploma*	27 (75)	14 (41)	
*High school diploma*	6 (17)	9 (27)	
*Education (years)*	13.7 ± 1.1	13.9 ± 1.3	*t* = -0.56, *p* = 0.57
*Employment*			χ^2^ = 0.42, *p* = 0.51
*Employee*	35 (97)	32 (94)	
*Unemployed*	1 (3)	2 (6)	
*Occupation*			χ^2^ = 6.09, *p* = 0.23
*Teaching*	17 (47)	17 (50)	
*Administration /Sales*	6 (17)	6 (18)	
*Computer science*	3 (8)	2 (6)	
*Accountancy*	5 (14)	2 (6)	
*Health*	0	4 (11)	
*Other*	4 (11)	2 (6)	
*Missing*	1 (3)	1 (3)	

Numbers represent *n* (%) or Mean ± SD

^a^Based on chi-square test or independent sample *t*-test where appropriate

In accordance with previous studies [44, 51], our results showed that overall, women rated their CBSEI outcome expectations significantly higher than their CBSEI self-efficacy expectations, both for the pre-post tests (139 ± 18 vs. 120 ± 25, *t* = 7.54, *p* < 0.001, respectively), as well as for the post-test (159 ± 18 vs. 147 ± 22, *t* = 5.82, *p* < 0.001, respectively).

When looking into the differences between the study groups, the pre-test scores of CBSEI self-efficacy expectations, CBSEI outcome expectations, knowledge, and attitudes were comparable (*t* = -1.07, *p* = 0.25; t = -0.85, *p* = 0.39; *t* = -0.15, *p* = 0.88; *t* = 1.62, *p* = 0.11, respectively; Table 3). Following childbirth education, both the intervention and control groups improved for all the measures (self-efficacy, attitudes, and knowledge; Table 3).

**Table 3:** Intervention and control within-group comparison (*n* = 70)

		***Control group (n* = *36)***			***Intervention group (n* = *34)***		
		*Pre-test*	*Post-test*	*paired t test*	*Pre-test*	*Post-test*	*paired t test*
CBSEI	Self-efficacy expectations	117 ± 23	139 ± 22	t = -6.03***	122 ± 28	155 ± 21	t = -7.95***
	Outcome expectations	137 ± 21	151 ± 18	t = -4.47***	141 ± 32	167 ± 13	t = -10.22***
Knowledge		56 ± 18	75 ± 14	t = -7.19***	56 ± 22	89 ± 13	t = -7.48***
Attitudes		4.6 ± 0.75	5.3 ± 0.79	t = -5.06***	4.3 ± 0.94	6.4 ± 0.54	t = -12.48***

Data are presented in Mean ± SD

^***^p < 0.001

**Fig. 2:**
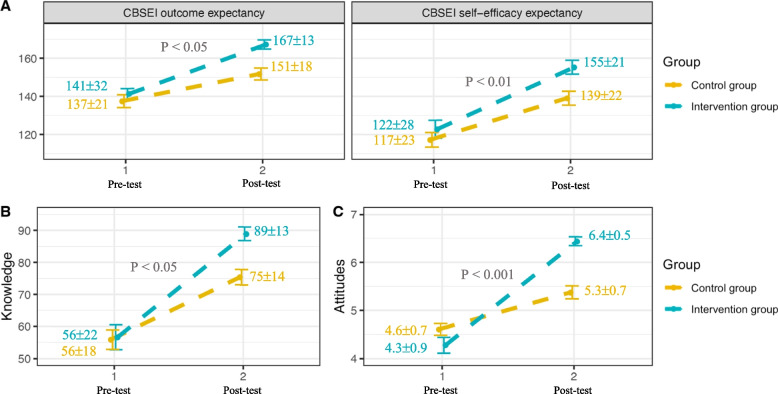
Graphical representation of the interaction effect between the study groups and changes in CBSEI outcome expectancy and CBSEI self-efficacy expectancy (**A**), knowledge (**B**) and attitudes (**C**) of upright positions and mobility during labor

The analysis revealed that the participants in the intervention group obtained significantly higher self-efficacy, attitudes, and knowledge post-test scores than those in the control group (Fig. 2). A two-way repeated measures ANOVA with a Greenhouse–Geisser correction confirmed significant interactions between the study groups in CBSEI self-efficacy change (F(1, 68) = 4.835, *p* < 0.05), with partial η_p_^2^ = 0.08 (medium effect size); CBSEI outcome expectations change (F(1, 68) = 8.232, *p* < 0.01), with partial η_p_^2^ = 0.11 (medium-large effect size); Attitudes change (F(1, 68) = 36.983, *p* < 0.001), with partial η_p_^2^ = 0.35 (large effect size); Knowledge change (F(1, 68) = 6.566, *p* < 0.05), with partial η_p_^2^ = 0.09 (medium effect size).

Since CBSEI self-efficacy has been shown to be strongly associated with labor outcomes, we evaluated how the participants’ attitudes and knowledge of upright positions and mobility during labor, and their sociodemographic characteristics impacted CBSEI self-efficacy. The analysis showed that CBSEI self-efficacy was significantly associated with the participants’ pre-test knowledge level of upright positions and mobility (Table 4; *r* = 0.406, *p* < 0.001); as well as with their pre-test attitudes toward upright positions and mobility (Table 4; *r* = 0.44, *p* < 0.001). Similarly, also at the post-test, CBSEI self-efficacy was associated with knowledge (*r* = 0.349, *p* < 0.001) and attitudes (*r* = 0.397, *p* < 0.001).

**Table 4:** Bivariate intercorrelations between the pre-test CBSEI self-efficacy, attitudes and knowledge scores of upright positions and mobility during labor, and the participants’ sociodemographic characteristics (*n* = 70)

Variable		1	2	3	4	5	6	7
CBSEI	1. Self-efficacy expectations	−						
	2. Outcome expectations	.547***	−					
3. Knowledge		.406***	.281*	−				
4. Attitudes		.334**	.189	.308**	−			
5. Age		-.102	.052	.063	0.005	−		
6. Education (years)		.068	-.013	-.023	.033	.107	−	
7. Family income		.132	.052	-.113	.003	.197	-.092	−

Data represents the Pearson r or Spearman r_s_

^*^p < 0.05

^**^p < 0.01

^***^p < 0.001

## Discussion

Childbirth education is an important intervention that may affect the labor and birth experience. Following the recent calls to provide evidence-based childbirth education practices [52, 53], this study focused on educational pedagogy to promote the use of upright positions and mobility during labor. To the best of our knowledge, our study is the first to show the significant positive impact of theory-based educational pedagogy in childbirth education. The current study demonstrates how inclusion of active learning activities to engage minds while participating in childbirth classes can support women to improve their knowledge, attitudes, and self-efficacy to be mobile and adopt comfortable positions of their choice, including upright positions. As a body of knowledge has shown that the level of perceived self-efficacy is strongly related to an individual's intention to perform a behavior [30, 31, 54], strengthened self-efficacy to be mobile and do take upright positions was our most important finding.

This study conceptualizes active learning experiences via the ICAP framework for cognitive engagement. Active learning is often described in terms of Kolb’s experiential learning cycle to postulate that concrete experiences are essential to provide a basis for learning [55]. In contrast to the common perception, concrete experiences, as suggested by the ICAP cognitive engagement theory, do not require hands-on participation but may be derived from minds-on engaging experiences [26, 27]. The cognitive engagement theory predicts that the more learners are cognitively engaged with their instructional experiences, the better their learning outcomes will be [56]. Consistent with previous studies [57], this study's findings demonstrated that learning with discovery-based and role-play activities followed by a collaborative dialogue, shifted women's cognitive engagement from passive to constructive and interactive modes. This, in turn, fostered women’s knowledge about upright labor positions and mobility significantly higher than that of the control condition where women learned with the passive cognitive engagement mode. This advantage of the ICAP framework for cognitive engagement to foster childbirth education has notable financial and accessibility benefits, suggesting that even without expensive educational technology or the need to change the curriculum, learning can be maximized.

Learning with the ICAP constructive and interactive cognitive engagement modes, as the current study's findings show, fostered not only knowledge levels, but also induced more positive attitudes and self-efficacy expectations toward the use of upright positions and mobility during labor, compared to the control group. Self-efficacy is a dynamic cognitive process in which the individual evaluates her/his capabilities to cope with different realities, influence events, and execute required behaviors [31, 58]. The Theory of Planned Behavior (TPB) suggests that both self-efficacy and attitudes toward the behavior strongly influence an individual’s behavioral intentions [30, 54]. From this, we may deduce that childbirth preparation which includes activities to induce cognitive engagement, might aspire women to have a stronger inclination to be more proactive consumers, willing to influence their labor process.

The findings demonstrate an overall significant association between knowledge, attitudes and self-efficacy expectations. Namely, higher knowledge levels and more positive attitudes were related to the development of stronger self-efficacy toward the ability to use upright positions and mobility during labor. This finding is in alignment with the Social Cognitive Theory which suggests that self-efficacy expectancies might be gained from personal experience with a situation or event, vicarious experience, or information about the experience [59]. Obviously, the majority of nulliparous women do not have previous personal childbirth experience to develop self-efficacy and therefore turn to childbirth preparation as the main source of their self-efficacy. This is true especially for the ultra-orthodox Jewish women participants in this study who do not have access to alternative sources of information such as online or television media sources.

### Strengths and limitations

Despite the advantages of our quasi-experimental study to compare the effectiveness of instructional approaches, a few limitations should be taken into consideration. To minimize diffusion between the intervention and control groups and to avoid any feelings of resentful demoralization among participants, we chose not to employ random assignment. This decision, however, could potentially introduce bias into our findings. To counteract this and mitigate potential bias, we employed a sequential recruitment strategy. Specifically, we filled each control group class (comprising up to 12 women) before recruiting for the intervention group, alternating in this manner throughout the recruitment process. Additionally, we conducted pretest evaluations of knowledge, attitudes, and self-efficacy to ensure that there were no significant initial differences between the groups. The study was conducted among a specific and homogenous community of ultra-orthodox Jewish women. Larger-scale studies that involve a more representative sample of the diverse public is recommended. In addition, the current study evaluated only self-efficacy and attitudes toward upright positions, further studies should involve an assessment of women's actual labor experience and their medical records to account for delivery outcomes. Finally, we evaluated only immediate knowledge, further studies should incorporate also a delayed post-test to assess the long-term effect. Therefore, these limitations warrant further studies to be conducted to validate the findings.

## Conclusion

There are two main practical implications to the study’s findings. First, childbirth self-efficacy can be modified by antenatal education. This makes childbirth education to be a crucial tool in supporting women-centered care. Second, childbirth education processes can be scaled up and maximized when constructive and interactive activities of cognitive engagement are involved.

This study evaluated in-person education; however, the findings can be relevant to online childbirth education as well. Since both in-person and online childbirth classes are predominantly based on one-way lectures, which according to the ICAP theoretical framework is a passive mode of engagement, this encourages minimal learning outcomes. We propose that this passive instructional approach can be shifted from passive to higher cognitive engagement mode by encouraging learners to generate new knowledge beyond what is provided (for example, drawing concept maps, taking notes in one’s own words); and by supporting debate or collaborative dialogue with other learners.

## Data Availability

Data are available upon request to the corresponding author.

## Abbreviations

ICAP framework: Interactive, Constructive, Active, Passive framework
CBSEI: The Childbirth Self-Efficacy Inventory

## References

[1] Lawrence A, Lewis L, Hofmeyr GJ, Styles C (2013). Maternal positions and mobility during first stage labour. Cochrane Database Syst Rev.

[2] Berta M, Lindgren H, Christensson K, Mekonnen S, Adefris M (2019). Effect of maternal birth positions on duration of second stage of labor: systematic review and meta-analysis. BMC Pregnancy Childb.

[3] Gupta JK, Sood A, Hofmeyr GJ, Vogel JP (2017). Position in the second stage of labour for women without epidural anaesthesia. Cochrane Database Syst Rev.

[4] Watson HL, Cooke A (2018). What influences women’s movement and the use of different positions during labour and birth: a systematic review protocol. Syst Rev.

[5] Zang Y, Lu H, Zhang H, Huang J, Ren L, Li C (2020). Effects of upright positions during the second stage of labour for women without epidural analgesia: a meta-analysis. J Adv Nurs.

[6] Gimovsky AC, Berghella V (2022). Evidence-based labor management: second stage of labor (part 4). Am J Obstetr Gynecol MFM.

[7] Deliktas A, Kukulu K (2018). A meta-analysis of the effect on maternal health of upright positions during the second stage of labour, without routine epidural analgesia. J Adv Nurs.

[8] Gönenç İM, Dikmen HA (2020). Effects of dance and music on pain and fear during childbirth. J Obstet Gynecol Neonatal Nurs.

[9] Akin B, YurteriTürkmen H, YalnızDilcen H, Sert E (2022). The effect of labor dance on traumatic childbirth perception and comfort: a randomized controlled study. Clin Nurs Res.

[10] WHO. WHO recommendations on intrapartum care for a positive childbirth experience. World Health Organization; 2018. https://www.who.int/publications/i/item/9789241550215. 30070803

[11] American College of Obstetricians and Gynecologists. ACOG committee opinion summary no. 687: approaches to limit intervention during labor and birth. 2017. https://www.acog.org/clinical/clinical-guidance/committee-opinion/articles/2019/02/approaches-to-limit-intervention-during-labor-and-birth?utm_source=redirect&utm_medium=web&utm_campaign=otn.

[12] Care Quality Commission. Maternity survey 2021. 2022. https://www.cqc.org.uk/publications/surveys/maternity-survey-2021 (Accessed 19/08/2022.

[13] Barasinski C, Debost-Legrand A, Lemery D, Vendittelli F (2018). Practices during the active second stage of labor: a survey of French midwives. Midwifery.

[14] Zang Y, Lu H, Zhao L, Zhang H, Hu Y, Fu L (2022). Barriers and facilitators to the implementation of a practice programme for upright positions in the second stage of labour in China: a qualitative study. Midwifery.

[15] Irvin L, De Leo A, Davison C (2022). Stand and deliver: an integrative review of the evidence around birthing upright. Br J Midwifery.

[16] Garbelli L, Lira V (2021). Maternal positions during labor: Midwives’ knowledge and educational needs in northern Italy. Eur J Midwifery.

[17] Thies-Lagergren L, Hildingsson I, Christensson K, Kvist LJ (2013). Who decides the position for birth? A follow-up study of a randomised controlled trial. Women Birth.

[18] Clews C, Church S, Ekberg M (2020). Women and waterbirth: a systematic meta-synthesis of qualitative studies. Women Birth.

[19] Kjeldsen LL, Dahlen HG, Maimburg RD (2022). Expectations of the upcoming birth–A survey of women’s self-efficacy and birth positions. Sexual Reprod Healthc.

[20] Campbell V, Nolan M (2019). ‘It definitely made a difference’: a grounded theory study of yoga for pregnancy and women's self-efficacy for labour. Midwifery.

[21] Lothian JA (2008). Childbirth education at the crossroads. J Perinat Educ.

[22] Chen I, Opiyo N, Tavender E (2018). Non-clinical interventions for reducing unnecessary caesarean section. Cochrane Database Syst Rev.

[23] Hong K, Hwang H, Han H (2021). Perspectives on antenatal education associated with pregnancy outcomes: systematic review and meta-analysis. Women Birth.

[24] Shand AW, Lewis-Jones B, Nielsen T (2022). Birth outcomes by type of attendance at antenatal education: An observational study. Austr N Z J Obstetr Gynaecol.

[25] Bonwell CC, Sutherland TE (1996). The active learning continuum: Choosing activities to engage students in the classroom. New Dir Teach Learn.

[26] Chi MTH, Wylie R (2014). The ICAP framework: linking cognitive engagement to active learning outcomes. Educ Psychol.

[27] Chi MTH, Adams J, Bogusch EB (2018). Translating the ICAP theory of cognitive engagement into practice. Cogn Sci.

[28] Quesnelle KM, Zaveri NT, Schneid SD (2021). Design of a foundational sciences curriculum: applying the ICAP framework to pharmacology education in integrated medical curricula. Pharmacol Res Perspect.

[29] Dubovi I (2019). Online computer-based clinical simulations: the role of visualizations. Clin Simul Nurs.

[30] Ajzen I (1991). The theory of planned behavior. Organ Behav Hum Decis Process.

[31] Bandura A (1977). Self-efficacy: toward a unifying theory of behavioral change. Psychol Rev.

[32] Dubovi I, Levy ST, Levy M, Zuckerman Levin N, Dagan E (2020). Glycemic control in adolescents with type 1 diabetes: are computerized simulations effective learning tools?. Pediatr Diabetes.

[33] Dubovi I, Adler I. The impact of COVID-19 induced anxiety on students’ engagement while learning with online computer-based simulations: the mediating role of boredom. Interactive Learning Environments 2022: 1–16.

[34] Williamson SP, Moffitt RL, Broadbent J, Neumann DL, Hamblin PS (2022). Coping, wellbeing, and psychopathology during high-risk pregnancy: a systematic review. Midwifery.

[35] Ministry of Health. High-Risk Pregnancy. 2023. https://www.health.gov.il/Subjects/pregnancy/during/Pages/highRisk_Pregnancy.aspx.

[36] The Israel Democracy Institute. Statistical Report on Ultra-Orthodox Society in Israel. 2020. https://en.idi.org.il/haredi/2020/?chapter=34273 (accessed 26.08.2022 2022).

[37] Engelsman SP, Huss E, Cwikel J (2018). How ultra-orthodox (Haredi) Israeli women cope with normative and difficult pregnancy and childbirth experiences. Nashim: J Jewish Women's Stud Gender Issues.

[38] OECD. Fertility rates 2022. https://data.oecd.org/pop/fertility-rates.htm.

[39] Korn L, Koren G, Yaakov A, Madar G, Blau A (2021). Evaluating the effectiveness of childbirth preparation courses on women’s self-efficacy among ultra-Orthodox Jewish religious women in Israel. Healthcare.

[40] Department of Health. Preparation for Birth and Beyond: A resource pack for leaders of community groups and activities. 2011. https://www.gov.uk/government/publications/preparation-for-birth-and-beyond-a-resource-pack-for-leaders-of-community-groups-and-activities.

[41] CAPEA. National competency standards for childbirth and early parenting educators Sydney Australia. 2018. https://capea.org.au/wp-content/uploads/2021/01/National-Competency-Standards-2019-inpageversion.pdf.

[42] Bruner J (1961). The act of discovery. Harv Educ Rev.

[43] Siccardi M, Valle C, Di Matteo F, Angius V (2019). A postural approach to the pelvic diameters of obstetrics: the dynamic external pelvimetry test. Cureus.

[44] Lowe NK (2000). Self-efficacy for labor and childbirth fears in nulliparous pregnant women. J Psychosom Obstet Gynecol.

[45] Lowe NK (1993). Maternal confidence for labor: Development of the childbirth self-efficacy inventory. Res Nurs Health.

[46] Bandura A (1982). Self-efficacy mechanism in human agency. Am Psychol.

[47] Pan W-L, Gau M-L, Lee T-Y, Jou H-J, Liu C-Y, Wen T-K (2019). Mindfulness-based programme on the psychological health of pregnant women. Women Birth.

[48] Taber KS (2018). The use of Cronbach’s alpha when developing and reporting research instruments in science education. Res Sci Educ.

[49] Desseauve D, Fradet L, Lacouture P, Pierre F (2017). Position for labor and birth: State of knowledge and biomechanical perspectives. Eur J Obstetr Gynecol Reprod Biol.

[50] Dagan E, Dubovi I, Levy M, Zuckerman Levin N, Levy ST (2019). Adherence to diabetes care: knowledge of biochemical processes has a high impact on glycaemic control among adolescents with type 1 diabetes. J Adv Nurs.

[51] Khaikin R, Marcus Y, Kelishek Y, Balik C (2016). The effect of childbirth preparation courses on anxiety and self-efficacy in coping with childbirth. Clin Nurs Stud.

[52] Downer T, Young J, McMurray A (2020). Are we still woman-centred? Changing ideologies, a history of antenatal education in Australia. Collegian.

[53] Levett K, Dahlen HG (2019). Perspective: childbirth education in Australia: have we lost our way?. Women Birth.

[54] Bosnjak M, Ajzen I, Schmidt P (2020). The theory of planned behavior: selected recent advances and applications. Eur J Psychol.

[55] Kolb DA (1984). Experience as the source of learning and development.

[56] Chi MT, Kang S, Yaghmourian DL (2017). Why students learn more from dialogue-than monologue-videos: analyses of peer interactions. J Learn Sci.

[57] Dubovi I, Lee VR (2019). Instructional support for learning with agent-based simulations: a tale of vicarious and guided exploration learning approaches. Comput Educ.

[58] Bandura A. Self-Efficacy. In: Craighead IBWWE, ed. The Corsini Encyclopedia of Psychology; 2010. p. 1–3. 10.1002/9780470479216.corpsy0836.

[59] Bandura A (1986). The explanatory and predictive scope of self-efficacy theory. J Soc Clin Psychol.

